# Identification phenotypic and genotypic characterization of biofilm formation in *Escherichia coli* isolated from urinary tract infections and their antibiotics resistance

**DOI:** 10.1186/s13104-019-4825-8

**Published:** 2019-12-05

**Authors:** Elnaz Davari Abad, Amin Khameneh, Leila Vahedi

**Affiliations:** 1 Department Of Microbiology, Faculty of Basic Science, Islamic Azad University, Urmia, Iran; 2 Student of Medicine, Faculty of Medicine, Tabriz University of Medical Sciences, Tabriz, Iran; 3 Liver and Gastrointestinal Disease Research Center, Tabriz University of Medical Sciences, Tabriz, Iran

**Keywords:** *Escherichia coli*, Urinary tract infection, *sfa* gene, *afa* gene, *foc* gene, Biofilm formation, Resistance antibiotics

## Abstract

**Objective:**

Urinary tract infections (UTIs) are the most common infectious diseases, and *Escherichia coli* is the most common pathogen isolated from patients with UTIs. The products of *sfa*, *afa and foc* genes are important for binding of the bacterium to urinary tract epithelium. Our aim was to investigate these genes in *E. colis* isolated from patients with UTIS. The frequencies of the genes were determined using PCR. Biofilm formation and antibiotic resistance rates were determined using microtiter plate and disk diffusion methods, respectively. The *P *<* 0.05* was considered statistically significant.

**Results:**

The frequencies of *sfa, afa and foc* were 75.3%, 17.5% and 22.5%, respectively showing a significantly higher prevalence of the *sfa gene*. The most effective antibiotics against the *E. colis* were nitrofurantoin and amikacin. The highest microbial resistance rates were also observed against amoxicillin and ampicillin. Furthermore, 12.7%, 6.3%, 74.7% and 6.3% of the isolates showed strong, moderate, weak capacities and no connections to form biofilms, respectively. The expression of the *sfa* gene was significantly associated with forming strong biofilms. Regarding the variabilities in the characteristics of *E. coli* strains associated with UTIs, it seems reasonable to adjust diagnostic and therapeutic methods according to the regional microbial characteristics.

## Introduction

Urinary tract infections (UTIs) are the most common human infectious disease affecting the bladder, kidneys and urinary tracts [1]. Overall, 150 million people are affected by UTIs worldwide [2, 3]. The incidence of UTIs is higher in women, and it has been estimated that 40–50% of adult women experience at least one UTI during their lifespans [1]. Kidney stones, diabetes, weak immune system can increase the risk of UTIs [3].

*Escherichia coli*, a Gram-negative bacterium, is responsible for more than 85% of all UTIs [4].

Biofilm formation ability is one of the important factors increasing the pathogenicity of bacteria and their resistance to antimicrobial agents. Biofilms are communities of microorganisms and their microbial products assisting bacteria to attach to uroepithelial cells. The products of *sfa, afa* and *foc* genes are particularly involved in these interactions [5]. In fact, the pili (Pap) and s fimbrial adhesion (*sfa*) which are encoded within the “operon” region of *sfa* gene confer resistance to uropathogenic bacteria against the host’s immune system and a wide range of antibiotics [3].

Particularly, some strains of *E. coli* called the extended—spectrum beta-lactamases (ESBL), have shown resistance to many antibiotics such as ampicillin and tetracycline [4–7]. The identification of these drug resistant microorganisms is essential for choosing proper antibiotics to avoid the waste of time and money and the development of multi-drug resistant bacteria [7, 8].

Polymerase Chain Reactions (PCR) now provides a sensitive and precise method for timely diagnosis of microbial infections. Furthermore, different genotypic and phenotypic detection methods (e.g. microtiter plate assays) are used to evaluate bacterial biofilm formation [6–8]. In the present study, we aimed to investigate the frequencies of *sfa*, *afa* and *foc* genes using PCR in *E. coli* isolated from patients with UTIs. We also assessed the ability of the isolated bacteria to form biofilms by microtiter plate assay and the tolerance rate of *E. colis* to antibiotics depending on specific genes.

## Main text

### Methods

#### Patients and collecting samples

This research was a cross-sectional research where 150 urine samples were gathered from patients with the symptoms of urinary tract infection who had been admitted at Amir Almomenin’s Hospital, Central Laboratory of the University of Medical Sciences, and Private and Medical Dinesh’s Laboratory through census method in Maragheh/Iran in 2018. To confirm infection with *E. coli*, the samples were cultured in the microbiology section in the EMB Agar and Blood Agar and were identified by the Gram, Indole, Citrate and MR-VP tests. Duplicate patient samples were excluded from this study. Finally, 79 samples for *E. coli* were recognized. The laboratory criteria of acute urinary tract infection with *E. coli* included one positive culture of colonies with a minimum number of 10^5^ colonies per 1 ml of urine [9]. This study was conducted on Azeri Turks, who are members of one of the largest ethnic groups in Iran [10].

#### Genotypic study

##### DNA extraction

In the current study, the boiling method was used for DNA extraction from the urine samples [11]. Specifically, several fresh bacteria colonies were mixed in 200 µl of buffer TE (Tris HCL 10 Mm + EDTA 1 Mm). Then, some water was boiled and after reaching the boiling point, the above sample was placed on a piece of unileet placed on the surface of water for boiling over 10 min. Finally, the sample was centrifuged at 10,000 rpm for 10 min and upper liquid was used for PCR.

##### PCR reaction

Specific primers were used to amplify the sequences of the *sfa*, *afa*, and *foc* genes [12, 13] (Table 1). As indicated in Table 1, the PCR assay was carried out in a total volume of 25 µl of mixture containing 22 µl master mix, 1 µl DNA sample, 1 µl forward primer, 1 µl reverse primer, and 0.2 µl tag polymerase. The PCR timetable program for *sfa*, *afa*, and *foc* genes is presented in Table 1.

**Table 1:** Primers and PCR timetable program for sfa, afa, and foc genes in UPEC strain

Genes	Initial cycle	Denaturation	Annealing	Extension	Final extension	Primers sequencing		Product size (bp)
sfa gene								
Time	4 M	30 S	30 M	40 S	3 M	F:5′ C C G T A A G A T G T C T G C G A G 3′	R:5′ A G C A A G T C T G G C A A C G A G 3′	100
Temperature	95	95	53	72	72			
Number of cycles	1	35	35	35	1			
afa gene								
Time	5 M	1 S	30 M	3 S	7 M	F:5′ G C T G G G C A G C A A A C T G A T A A C T C T C 3′	R:5′ C A T C A A G C T G T T T G T T C G T C C G C C G 3′	750
Temperature	95	95	60	72	72			
Number of cycles	1	35	35	35	1			
foc gene								
Time	4 M	1 S	1 M	2 S	10 M	F:5′ G G T G G A A C C G C A G A A A A T A C 3′	R:5′ G A A C T G T T G G G G A A A G A G T G 3′	388
Temperature	95	95	58	72	72			
Number of cycles	1	35	35	35	1			

Once analyzed by 2% agarose gel electrophoresis, the PCR products were stained with ethidium bromide and photographed.

##### Assessment of biofilm formation via phenotype method

For investigating the ability of *E. coli* isolates to produce biofilms, biofilm test was performed in laboratory based on the Microtiter Plates Assay as follow as:

The microtiter plate method was used for evaluating the formation of UPEC biofilm. The Microtiter Plates Assay method, such as ELISA, the color-producing chromogen in this technique is fuchsin, whose color intensity is directly related to the concentration of biofilm.

Initially, one loop full of bacteria colony was infused into one tube, including 5 ml nutrient broth where this tube was heated at 37 °C for 18 to 24 h. Then, 1 ml of bacterial suspension was injected into the tube, including 10 ml sterile nutrient broth, with this tube being heated at 37 °C for a duration of 18 to 24 h. Regarding the injection only the sterile culture environment of nutrient broth into control well, microtiter plate was heated at 37 °C for 24 h. After the draining and washing of the wells three times by a sterile physiology serum, the plates were vigorously shaken to eliminate the disconnected cells. For stabilizing the cells, 200 µl ethanol 96% was added to wells. After 15 min, the wells were drained, dried and stained by 200 µl fuchsin for 5 min. After 5 min, the wells were washed slowly by urban water and were filled with 200 µL acetic acid 33% as solvent. After plate incubation for 15 min at 37 °C, the Optical Density of the wells stained with fuchsin was screened by ELISA at a wavelength 492 nm. All measurements were repeated three times and the culture environment was used as negative control. A standard deviation larger than the negative control optical absorption was used as Cut-off. The ability of biofilm formation was calculated using the following formulas [14] that shown in the table. In the current study, OD ≥ 0.1, 0.07 ≤ OD ≤ 0.09, 0.01 ≤ OD ≤ 0.06 and OD ≤ 0.009 were considered strong, average, weak and no connection, respectively.

##### Assessment of antibiotic resistance in *E. coli*

The antibiotic sensitivity was examined by Kirby Beuer method (disk diffusion) [14]. The diameter of the zones of inhibition was measured by a ruler in millimeter. In this research, the antibiotics utilized against *E. coli* pathogens included Imipenem, Ciprofloxacin (cp5), Tobramycin (TOB10), Ampicillin, Tetracycline (TE30), Amikacin (AN30), Amoxicillin (AMX25), Nalidixic Acid (NA30), Nitrofurantoin, Cefepime, Gentamycin (GM10), Ceftazidime (CAZ30), Chloramphenicol, and Ceftriaxone (CRO30).

#### Data analysis

Data were analyzed using SPSS 23 for the frequency and percentage. The comparison between variables was analyzed by a Chi square or Fisher’s exact tests. *P *<* 0.05* was considered statistically significant.

### Results

In this study, the PCR reaction was done on 79 samples from urine specimens with UTI symptoms and suspected to have *E. coli,* after confirmation (Additional file 1: Fig. S1).

The PCR results indicated that the highest and lowest frequency of fimbrial genes was associated with *sfa* and *sfa*-*afa*-*foc* genes (Table 2). Then, the frequency of *sfa* gene was compared to other genes. There was a significant difference between *sfa* with *afa*, *foc*, *sfa*-*afa*, *sfa*-*foc*, *afa*-*foc,* and *sfa*-*afa*-*foc* with 0.04, 0.001, 0.009, 0.001 and 0.00 P-values, respectively.

**Table 2:** The characteristics of genes of sfa, afa, and foc

Degrees of biofilm						Resistance to antibiotics													
Genes	Frequency (%)	Strong	Moderate	Weak	No connection	Amoxicillin	Tetracycline	Ciprofloxacin	Cefepime	Ceftazidime	Ceftriaxone	Tobramycin	Gentamycin	Nitrofurantoin	Amikacin	Ampicillin	Chloramphenicol	Imipenem	Nalidixic acid
sfa	27 (34.18)	4 (14.8)	0 (0)	21 (77.8)	2 (7.4)	20 (33.33)	14 (32.56)	12 (40)	5 (31.25)	4 (23)	9 (31.03)	0 (0)	4 (33.33)	0 (0)	2 (22.22)	20 (34.48)	0 (0)	3 (37.5)	16 (41.03)
afa	14 (17.72)	0 (0)	2 (14.3)	12 (58.7)	0 (0)	13 (21.67)	10 (23.26)	3 (10)	4 (25)	2 (11.76)	5 (17.42)	2 (50)	3 (25)	1 (33.33)	1 (11.11)	11 (18.97)	2 (40)	1 (12.50)	6 (15.38)
foc	18 (22.78)	3 (11.1)	1 (5.6)	13 (72.2)	1 (5.6)	14 (23.33)	10 (23.26)	8 (26.67)	4 (25)	6 (35.29)	9 (31.03)	1 (25)	2 (16.67)	1 (33.33)	3 (33.33)	14 (24.14)	2 (40)	2 (25)	8 (20.51)
sfa,afa	3 (3.80)	0 (0)	0 (0)	3 (100)	0 (0)	2 (3.33)	1 (2.33)	1 (3.33)	0 (0)	0 (0)	0 (0)	0 (0)	1 (8.33)	0 (0)	0 (0)	2 (3.45)	0 (0)	0 (0)	2 (5.13)
sfa,foc	11 (13.92)	3 (11.1)	1 (9.1)	6 (54.5)	1 (9.1)	6 (10)	6 (13.95)	5 (16.67)	2 (12.50)	4 (23.53)	3 (10.34)	0 (0)	1 (8.33)	0 (0)	2 (22.22)	7 (12.07)	0 (0)	1 (12.5)	6 (15.38)
afa,foc	5 (6.33)	0 (0)	1 (20)	3 (60)	1 (20)	4 (6.67)	2 (4.65)	1 (3.33)	1 (62.5)	1 (5.88)	3 (10.34)	1 (25)	1 (8.33)	1 (33.33)	1 (11.11)	3 (5.17)	1 (20)	1 (12.5)	1 (2.56)
sfa,afa foc	1 (1.27)	0 (0)	0 (0)	1 (100)	0 (0)	1 (1.67)	0 (0)	0 (0)	0 (0)	0 (0)	0 (0)	0 (0)	0 (0)	0 (0)	0 (0)	1 (1.72)	0 (0)	0 (0)	0 (0)
Total	79 (100)	10 (12.7)	5 (6.3)	59 (74.7)	5 (6.3)	60 (100)	43 (100)	30 (100)	16 (100)	17 (100)	29 (100)	4 (100)	12 (100)	3 (100)	9 (100)	58 (100)	5 (100)	8 (100)	37 (100)

The total of items were considered as frequency (percentage)

#### Results of microtiter plate biofilm formation

Among 79 *E. coli* isolates from urinary tract infections, 10 isolates (12.7%), 5 isolates (6.3%), 59 isolates (74.7%) and 5 isolates (6.3%) showed strong, moderate, weakly and no connection biofilm formation ability, respectively.

The capacity of biofilm formation were compared between genes, where there was no significant correlation between them; however, the strongest and the weakest had related to *sfa* and *afa* genes, respectively (Table 2). Of note, *afa* even has reduced ability of biofilm formation in combination with other genes.

In the antibiotic resistance, the most resistant strains were related to the amoxicillin and ampicillin antibiotics, while the greatest sensitivity was associated with nitrofurantoin and amikacin (Tables 2 and 3).

**Table 3:** Results of antibiogram for genes of UPEC strain

Antibiotics	Resistant*	Intermediate*	Sensitive*
Amoxicillin	60 (18.13)	4 (2.21)	15 (2.53)
Tetracycline	43 (12.99)	9 (4.97)	27 (4.55)
Ciprofloxacin	30 (9.06)	9 (4.97)	40 (6.73)
Sefipem	16 (4.83)	15 (8.29)	48 (8.08)
Ceftazidime	17 (5.14)	7 (3.87)	55 (9.26)
Ceftriaxone	29 (8.79)	7 (3.87)	43 (7.24)
Tobramycin	4 (1.21)	28 (15.47)	47 (7.91)
Gentamicin	12 (3.63)	14 (7.73)	53 (8.92)
Nitrofurantoin	3 (0.91)	7 (3.87)	69 (11.62)
Amikacin	9 (2.72)	30 (16.57)	40 (6.73)
Ampicillin	58 (17.52)	10 (5.52)	11 (1.85)
Chloramphenicol	5 (1.51)	8 (4.42)	66 (11.11)
Imipenem	8 (2.42)	25 (13.81)	46 (7.74)
Nalidixic Acid	37 (11.18)	8 (4.42)	34 (5.72)

*The total of items were considered as frequency (percentage)

### Discussion

*E. coli* is the most common causative agent of UTIs in both outpatients and inpatients. If left untreated, UTIs may culminate in serious consequences such as renal failure. Pyelonephritis usually develops following a simple bladder infection (i.e. cystitis) [5]. The ability of pathogenic bacteria to adhere to the urinary tract epithelium using pili (fimbriae) is the most important pathogenic feature leading to UTIs [15]. Various genes encoding pili can be identified by molecular techniques such as PCR [16].

Our results revealed a relatively high frequency of *sfa* gene compared with the *afa* and *foc* in *E. coli* strains isolated from patients with UTIs. This probably indicates the essential role of this gene in the development of UTIs. Various studies have reported the role of *sfa* gene in encoding adherence molecules involved in the pathophysiology of pyelonephritis caused by *E. coli* [3]. In line with our finding, Jalali et al. [17] reported that 32% of UTIs patients expressed the *sfa* gene. In another study among children suffering from UTI caused by *E. coli*, a high frequency of the *sfa* gene was reported [18]. Likewise, the frequency of the *afa* gene reported in another study was similar to the present report [19].

In the present study, strong and weak biofilm-forming capabilities were associated with the expressions of the *sfa* and *afa* genes, respectively. Reduction of biofilm formation ability was observed in combination of *afa* gene with other genes.

According to a study conducted by Lane MC et al., fimbria I molecule is upregulated during UTIs to ensure bacterial motility. This phenomenon is particularly important during 24 h after the colonization of bacteria in the bladder [20]. In another study, Goetz et al. [21] described the role of *papG* gene in augmenting the ability of bacteria to connect to surfaces. In the report of Ponnusamy et al. [22], only 23.6% of *E. coli* strains were able to from strong biofilms. These reports highlight the roles of *P fimbriae* (*pap*), *afa*, *hemolysin* (*hly*), and *sfa/foc* in biofilm formation by bacteria causing human infections. These molecules help bacteria to colonize and damage tissues and debilitate the host’s defense mechanisms which ultimately lead to clinical manifestations [23].

In the present study, the highest resistance rates of the isolated *E. coli* strains were observed against amoxicillin and ampicillin. This was while the greatest sensitivities were related to nitrofurantoin and amikacin antibiotics. In the study of Gazmoh et al. in Ethiopia, Adimi et al. in Nigeria and Abdollahi et al. in Tehran, *E. coli* strains from patients with UTIs demonstrated a similar resistance pattern to the present study [24–26].

*Escherichia coli* is considered as an important cause of UTI in patients referring to health centers. The severity of the infection depends on both the host’s immune competency and the distribution of virulence factors among pathogenic bacteria. UPEC isolates are genetically heterogeneous bacteria which have variable capacities for biofilm formation, colonization, invasion, and proliferation in the urinary tract [27, 28].

In another study by Jha et al. [29] on 244 patients with UTIs in Japan, *E. coli* was reported as the most frequent causative agent, and the least resistance rate was related to ciprofloxacin. In contrary to our results; however, Keikha et al. [30] in their study on 87 urinary *E. coli* isolates reported the highest antibiotic resistance against cotrimoxazole. These inconsistencies can be related to parameters such as sample sizes, the accuracies of sampling and testing methods, as well as different geographical locations [27–30].

Generally, *E. coli* strains causing UTIs represent increasing rates of antibiotic resistance, especially against the first (e.g. ampicillin) [31] the third (e.g. cephalosporins and aminoglycosides) generations of broad-spectrum antibiotics [32]. These high rates of antibiotic resistance may be due to the unprescribed availability and uncontrolled usage of antibiotics, especially in developing countries. Therefore, selecting antibiotics for treating bacterial infections should be according to the results of urine culture, antibiotic susceptibility, and biofilm formation analyses.

### Conclusion

There were differences between the characteristics of UPEC in this area and different regions in terms of frequency, formation of biofilm, and drug resistance. These differences were even observed among strains. By collecting the characteristics of UPEC strains in each region, the epidemiological characteristics of native isolates were distinguished. Therefore, it is possible to diagnose this condition earlier and offer appropriate treatments.

## Limitation

This study was limited by short duration and moderate sample. Ultimately, it is recommended to performing of the other studies on other genes between control and case groups.

## Supplementary information

**Additional file 1: Fig S1.** *Escherichia coli* strains including of *sfa, afa* and *foc* genes on agarose gel.

## Data Availability

The datasets used and analyzed during the current study are available from the corresponding author and first author on not allowed from the centers. Private and Medical Dinesh’s Laboratory in Maragheh and Liver and Gastrointestinal Disease Research Center of Tabriz University of Medical Sciences are where supporting our findings.

## Abbreviations

*E. coli*: *Escherichia coli*
UTIs: urinary tract infections
UPEC: uropathogenic *Escherichia Coli*
*Pap*: pyelonephritis associated pili
*sfa*: s fimbrial adhesion
OD: optical density
NM: nanometer
µL: microliter
ML: milliliter
C: centigrade
