# Supplementary figures and images for: Identification phenotypic and genotypic characterization of biofilm formation in Escherichia coli isolated from urinary tract infections and their antibiotics resistance
Source: BMC Res Notes. 2019 Dec 5;12:796. doi: 10.1186/s13104-019-4825-8 (PMC6896667; doi:10.1186/s13104-019-4825-8)

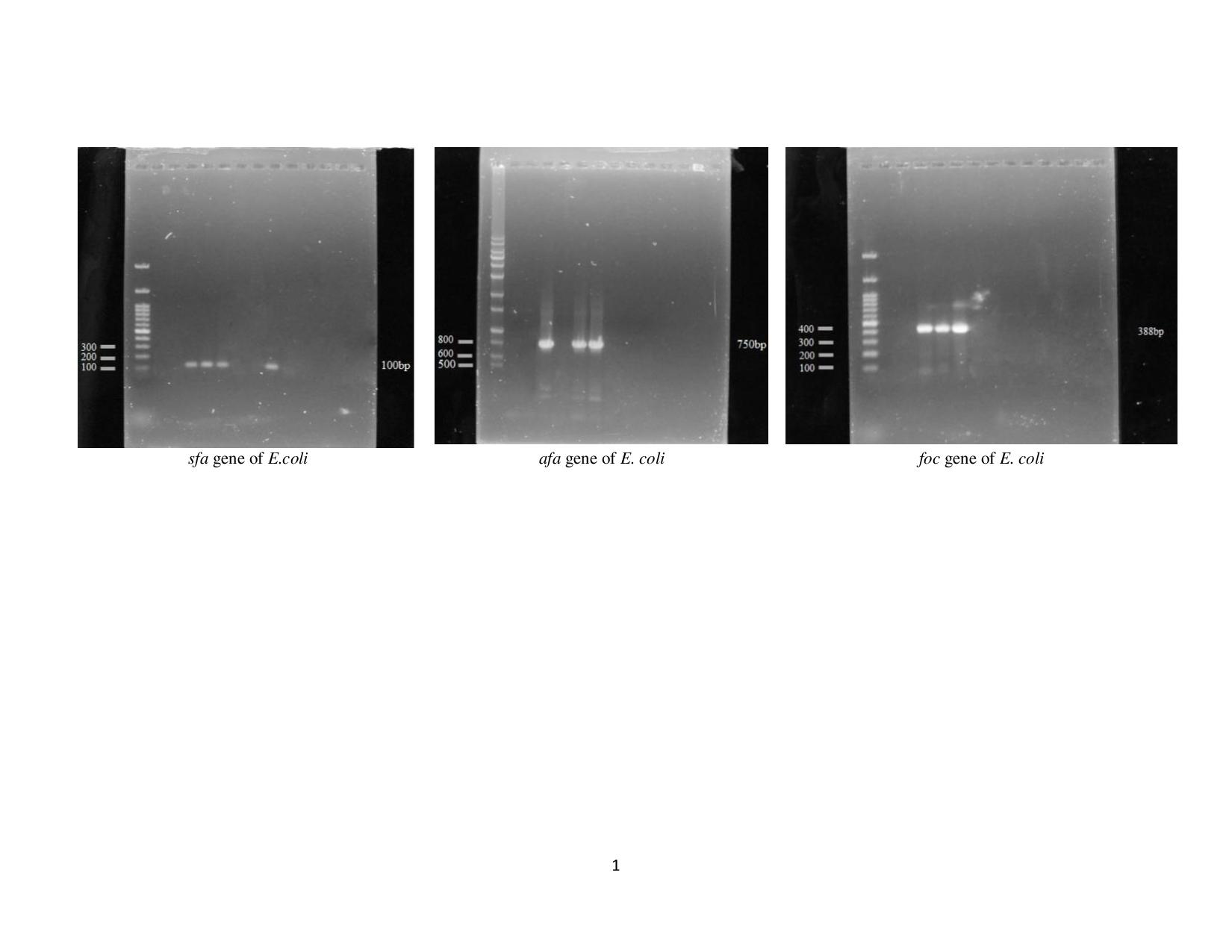

Supplement: Supplementary file 1 — Additional file 1: Fig S1. Escherichia coli strains including of sfa, afa and foc genes on agarose gel. [file 13104_2019_4825_MOESM1_ESM.jpg]
